# *Pseudomonas aeruginosa* Exploits Lipid A and Muropeptides Modification as a Strategy to Lower Innate Immunity during Cystic Fibrosis Lung Infection

**DOI:** 10.1371/journal.pone.0008439

**Published:** 2009-12-23

**Authors:** Cristina Cigana, Laura Curcurù, Maria Rosaria Leone, Teresa Ieranò, Nicola Ivan Lorè, Irene Bianconi, Alba Silipo, Flora Cozzolino, Rosa Lanzetta, Antonio Molinaro, Maria Lina Bernardini, Alessandra Bragonzi

**Affiliations:** 1 Infection and Cystic Fibrosis Unit, San Raffaele Scientific Institute, Milano, Italy; 2 Dipartimento di Biologia Cellulare e dello Sviluppo, Sapienza-Università di Roma, Roma, Italy; 3 Dipartimento di Chimica Organica e Biochimica, Università di Napoli Federico II, Napoli, Italy; CEINGE Biotecnologie Avanzate, Napoli, Italy; 4 Istituto Pasteur-Fondazione Cenci Bolognetti, Roma, Italy; Universidad Nacional, Costa Rica

## Abstract

*Pseudomonas aeruginosa* can establish life-long airways chronic infection in patients with cystic fibrosis (CF) with pathogenic variants distinguished from initially acquired strain. Here, we analysed chemical and biological activity of *P. aeruginosa* Pathogen-Associated Molecular Patterns (PAMPs) in clonal strains, including mucoid and non-mucoid phenotypes, isolated during a period of up to 7.5 years from a CF patient. Chemical structure by MS spectrometry defined lipopolysaccharide (LPS) lipid A and peptidoglycan (PGN) muropeptides with specific structural modifications temporally associated with CF lung infection. Gene sequence analysis revealed novel mutation in *pagL*, which supported lipid A changes. Both LPS and PGN had different potencies when activating host innate immunity via binding TLR4 and Nod1. Significantly higher NF-kB activation, IL-8 expression and production were detected in HEK293hTLR4/MD2-CD14 and HEK293hNod1 after stimulation with LPS and PGN respectively, purified from early *P. aeruginosa* strain as compared to late strains. Similar results were obtained in macrophages-like cells THP-1, epithelial cells of CF origin IB3-1 and their isogenic cells C38, corrected by insertion of cystic fibrosis transmembrane conductance regulator (CFTR). In murine model, altered LPS structure of *P. aeruginosa* late strains induces lower leukocyte recruitment in bronchoalveolar lavage and MIP-2, KC and IL-1β cytokine levels in lung homogenates when compared with early strain. Histopathological analysis of lung tissue sections confirmed differences between LPS from early and late *P. aeruginosa*. Finally, in this study for the first time we unveil how *P. aeruginosa* has evolved the capacity to evade immune system detection, thus promoting survival and establishing favourable conditions for chronic persistence. Our findings provide relevant information with respect to chronic infections in CF.

## Introduction

The strategy of innate immune recognition is based on the detection of constitutive and conserved products of microbial metabolism called pathogen-associated molecular patterns (PAMPs) or alternatively microbe-associated molecular patterns (MAMPs), since they are also common to all microbe and not only to the pathogen version [1]. These molecular signatures are recognized by the host through a family of pattern recognition receptors (PRRs), which includes Toll-like (TLRs) and nucleotide binding and oligomerization domain-like receptors NLR (Nod-Like receptor) [2]. For example, lipid A contained in bacterial lipopolysaccharide (LPS) acts as a PAMP and is sensed by the cognate PRR TLR4-MD2, while different motifs contained in peptidoglycan (PGN) of Gram-positive or Gram-negative bacteria are recognized by the intracellular PRRs Nod1 and Nod2 [3], [4]. Interaction of PAMPs with PRRs results in activation of antimicrobial responses [1], such as production of antimicrobial peptides and secretion of pro-inflammatory cytokines, necessary for pathogen's eradication. However, many pathogens have evolved adaptive strategies for subverting the host innate immune system by evading detection by PRRs and/or impairing the downstream cellular signalling pathway [5]. In this study, we present evidence that *Pseudomonas aeruginosa* exploits PAMPs modification as a strategy to lower innate immune system detection and signalling during chronic stages of lung infection in fibrosis cystic (CF) patients.

CF lung disease is characterized by transient airway *P. aeruginosa* infections and excessive neutrophil-dominated inflammation early in life followed by permanent chronic infection that causes persistent respiratory symptoms and decline in lung functions [6]. The long term colonization of CF airways selects pathoadaptive variants with several features which differentiate late *P. aeruginosa* isolates from the initially acquired strain [7], [8]. *P. aeruginosa* strains that initiate infections are characterized by a large arsenal of virulence factors, such as many toxic factors, like pyocyanin, a type III secretion system (T3SS), several proteases, lipases and phospholipases, rahmnolipids and other factors [9]. In contrast, these invasive functions are selected against in CF chronic infection leading to less virulent but more persistent phenotypes including alginate producing mucoid strains [7]. However, besides all these relevant features, which contribute to either forms of infection, the LPS modification appears to be one of the main factors in the adaptation of this pathogen during chronic infections. It is already known that *P. aeruginosa* acute infection also implies a consistent change in LPS lipid A structure [10] even though no conclusive information are present on the putative LPS lipid A changes in the acute to chronic evolution of the infection. In general, production of fully hexa-acylated lipid A is associated with a more strong TLR4 mediated inflammatory response while lipid A with lower levels of acylation triggers reduced cellular responses [11]. However, lipid A isolated from clinical strains of *P. aeruginosa* often results in a blend of different species [10] with a resulting biological activity that might be different when compared to single species bioactivity. Lipid A modifications are regulated by the environmental sensor-kinase transcriptional regulatory system PhoP-PhoQ and catalysed by PagP, PagL and LpxO which lead to acylation, deacylation and hydroxylation of the molecule, respectively [12], [13], [14].

As for PGN, an essential cell wall component, no data have been produced so far on the structure itself and eventual alterations in infection by *P. aeruginosa*. PGN changes may even more advantage bacterial life within the host and contribute to establish successful chronic infection in CF patients. In fact, in these last years a growing body of studies are highlighting the role played by muropeptide receptors Nod1 and Nod2 in the development of both antibacterial responses and chronic inflammation, such as Crohn's disease and asthma [15]. To fill the gap in the context of CF disease mediated by *P. aeruginosa* infection we analysed, at chemical and biological level, the LPS and PGN from three clonal strains of one *P. aeruginosa* lineage. *P. aeruginosa* strains were isolated at the onset of infection and after years of chronic colonization from a CF patient with a severe course of the airways infection [8]. Our results showed that the biological activity of LPS and PGN is consistent with a reduced immunostimulatory potential in accordance with the need to evade immune system and favour survival during the course of the chronic *P. aeruginosa* infection.

## Results

### *P. aeruginosa* Lipid A Chemical and Genetic Modifications in Clonal Strains of Early Colonization and Late Chronic Infection of CF Patient

The LPS of *P. aeruginosa* sequential strains AA2, isolated at the onset of chronic colonization, and AA43 (mucoid) and AA44, isolated before patient's death (Figure S1), was extracted by conventional methods (see Supporting Information File S1) and showed the typical ladder like pattern by SDS-PAGE electrophoresis (data not shown). The O-repeating unit resulted identical in all three strains and the structure is already reported elsewhere [16].

**Figure 1:**
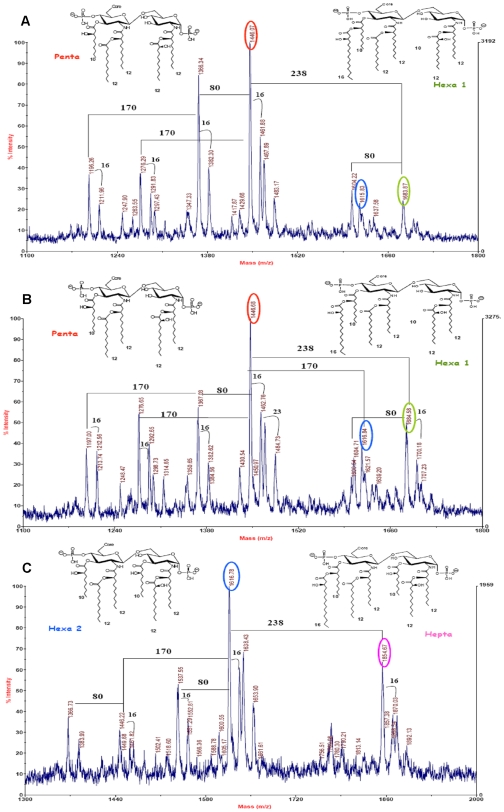
Chemical structure of *P. aeruginosa* lipid A. MALDI MS spectra of lipid A blend obtained by acid hydrolysis of LPS from *P. aeruginosa* clinical strains isolated at the onset of chronic colonization (AA2) (**A**) and after years of chronic infection (AA43 and AA44) from a CF patient (**B** and **C**). A difference of 238 Da corresponds to a 16∶0 fatty acid residue whereas 170 corresponds to a 10∶0 (3-OH) residue and 80 Da are indicative of a phosphate group. The 16 Da difference is relative to the presence of a Hydroxy group at C-2 of the secondary 12∶0 fatty acids. The non indicated ion peaks are relative to the species already indicated and bearing sodium or potassium counter-ions.

The structure of LPS lipid A of the three *P. aeruginosa* strains (Figure 1) was determined by chemical analyses and MS spectrometry (see Supporting Information File S1 for the whole structure determination). As shown in Figure 1, MALDI negative ion spectra revealed that LPSs extracted from AA2 and AA43 presented the same lipid A species even though with a different relative abundance. In both cases, MALDI mass spectra showed a main peak that matched with a penta-acylated lipid A constituted by a *bis*-phosphorylated disaccharide backbone carrying a 10∶0 (3-OH) in ester linkage on GlcN II and two 12∶0 (3-OH) in amide linkage on both GlcN residues. Furthermore, both 12∶0 (3-OH) were substituted by a secondary fatty acid, a 12∶0 on GlcN II and a 12∶0 (2-OH) on the GlcN I. Both lipid A blend also contained a tetra-acylated lipid A deriving from the previous by loss of the only 10∶0 (3-OH) in ester linkage on GlcN II. An asymmetric hexa-acylated lipid A bearing the extra 16∶0 that esterifies the 10∶0 (3-OH) on GlcN II was present almost exclusively in the lipid A blend of strain AA43. Minor species carrying two secondary 12∶0 (2-OH) fatty acids and others lacking a phosphate group were also present.

As for AA44 completely different lipid A were found, in which the main difference with those above was the presence of a further 10∶0 (3-OH) on GlcN I. The most prevalent one was a symmetric hexa-acylated lipid A constituted by a *bis*-phosphorylated disaccharide backbone carrying two 10∶0 (3-OH) in ester linkage and two 12∶0 (3-OH) in amide linkage. Further, both amide chains were substituted by a secondary fatty acid, a 12∶0 and a 12∶0 (2-OH) on the GlcN II and I, respectively. In addition, hepta-acylated lipid A bearing the additional 16∶0 on GlcN II species was present.

Lipid A modifications in AA2, AA43 and AA44 were stable as they were not lost after serial passages *in vitro*, which would suggest the presence of genetic mutations. Sequence analysis and multiple alignment of *phoP*, *phoQ*, *pagL*, *lpxO1* and *lpxO2* genes responsible for modifying lipid A, revealed *pagL* mutation in AA44 but not in AA2 and AA43 strains (Table S1 in Supporting Information File S1 and Figure S2). In particular, insertion of four nucleotides (CCTG) at position 502 lead to a frameshift mutation.

### LPS of *P. aeruginosa* Clinical Isolates Stimulates Different Inflammatory Response in Human Cells (HEK 293-hTLR4/MD2-CD14, C38 and THP-1) Including Those of CF Origin (IB3-1)

**Figure 2:**
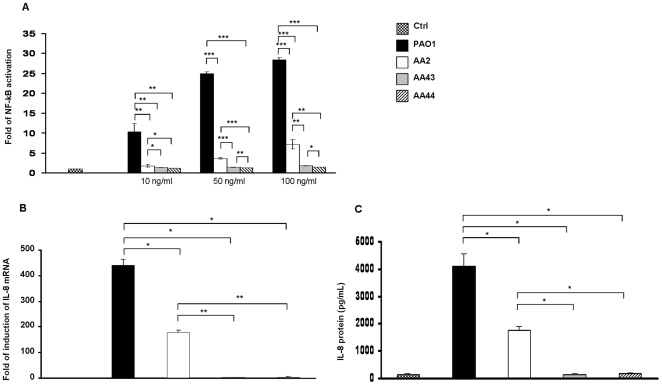
Stimulation of HEK 293-hTLR4/MD2-CD14 with LPS derived from the three clinical isolates of *P. aeruginosa* AA2, AA43 and AA44. **A**) Fold of activation of NF-kB after 4 h of stimulation with different concentrations of LPS; commercial LPS of *P. aeruginosa* was used as a control. **B**) IL-8 mRNA induction after stimulation with 100 ng/mL of LPS for 4 h. **C**) IL-8 secretion after stimulation with 100 ng/mL of LPS for 24 h. Commercial LPS of PAO1 was used as a control. *p<0.05, **p<0.01, ***p<0.001 in the Student's t-test.

HEK 293-hTLR4/MD2-CD14 were exposed to different LPSs concentration (10, 50 and 100 ng/mL) of *P. aeruginosa* AA2, AA43, AA44 and PAO1 for 4 h and NF-kB activation was evaluated through the assessment of luciferase activity (Figure 2A). LPS of *P. aeruginosa* AA2 induced a significantly higher NF-kB activation with respect to cells exposed to LPSs of AA43 and AA44 (LPS AA2 vs AA43 and AA44 p<0.01) in a dose dependent manner. IL-8 expression was measured through real time quantitative (q)-PCR after 4 h stimulation, as above (Figure 2B). In accordance with NF-κB activity, the level of IL-8 expression increased higher after stimulation with LPS from AA2 with respect to LPSs from AA43 and AA44 (LPS AA2 vs AA43 and AA44 p<0.01). For ELISA assay, HEK 293-hTLR4/MD2-CD14 were stimulated for 24 h with *P. aeruginosa* LPS as above (Figure 2C). The level of IL-8 release induced by LPS from AA2 was significantly higher than that induced by LPSs of AA43 and AA44 (LPS AA2 vs AA43 and AA44 p<0.05). Under all conditions, stimulation with LPS from PAO1 was considerably higher than those induced by LPSs from the clinical isolates (LPS PAO1 vs AA2, AA43 and AA44 p<0.05) (Figure 2A, B and C).

**Figure 3:**
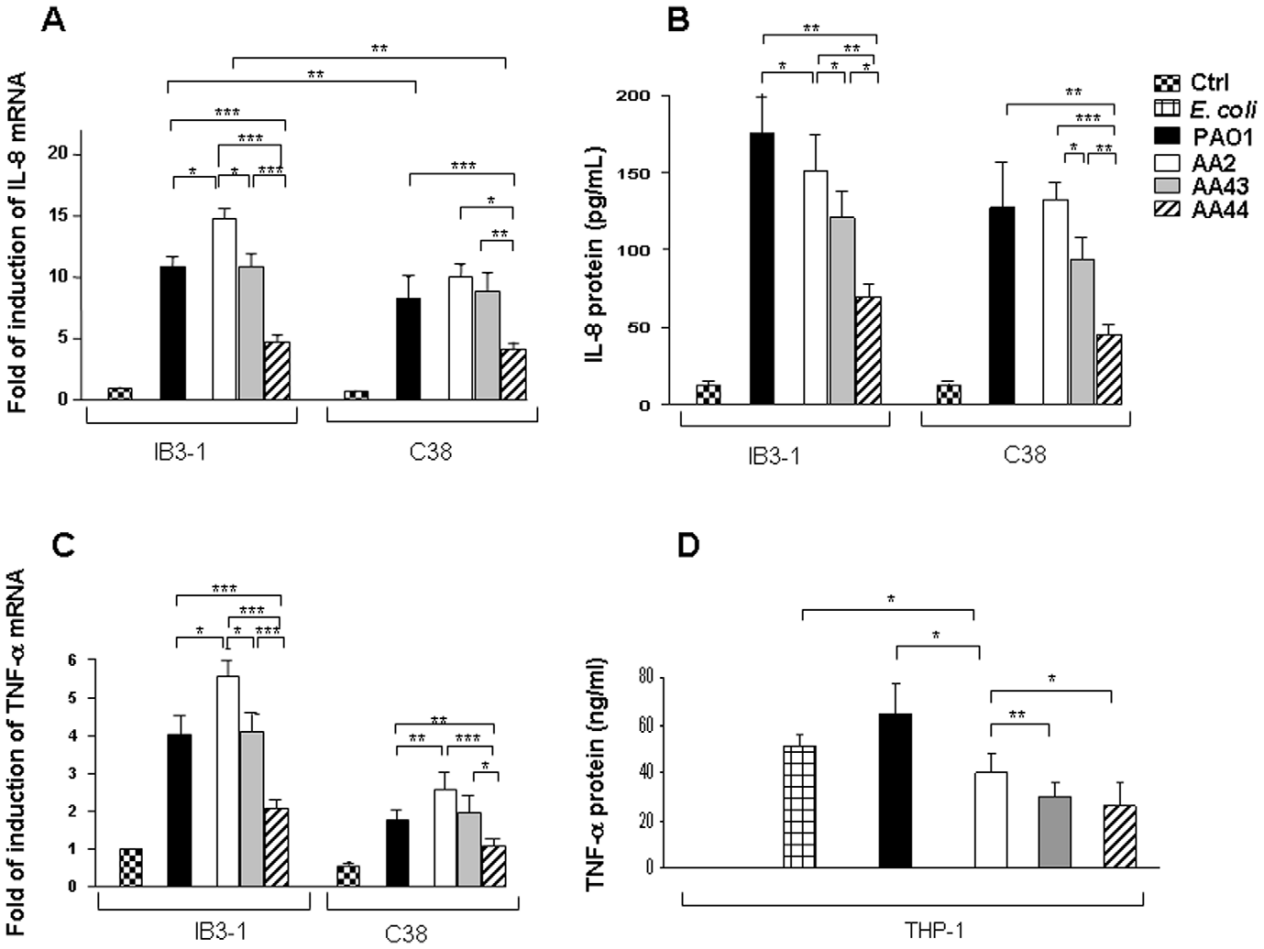
Response of IB3-1, C38 and THP-1 cells after stimulation with LPS derived from the three clinical isolates of *P. aeruginosa* AA2, AA43 and AA44. **A**) Fold of induction of IL-8 and **C**) TNF-α mRNA in IB3-1 and C38 cells after stimulation with 100 ng/mL of LPS for 4 h. Commercial LPS of PAO1 was used as control. The values represent the expression levels relative to untreated IB3-1 (means±SD). **B**) IL-8 secretion from IB3-1 and C38 cells after stimulation with 100 ng/mL of LPS for 24 h. LPS of PAO1 was used as control. **D**) TNF-α secretion from THP-1 after stimulation for 6 h with 100 ng/mL of LPS derived from the clinical isolates AA2, AA43 and AA44. LPSs from *P. aeruginosa* serotype 10^22^ and from *E. coli* serotype OIII:B4 (Sigma) were used as controls. *p<0.05, **p<0.01, ***p<0.001 in the Student's t-test.

Next, we tested CF respiratory cells (IB3-1) and the isogenic corrected cells (C38) after LPS stimulation. When tested for the pro-inflammatory markers, in IB3-1 cells LPS from AA2 induced significantly more IL-8 and TNF-α expression, assessed by real time q-PCR, when compared with LPSs from AA44 and AA43 (LPS AA2 vs AA43 and AA44 p<0.05) (Figure 3A and C). IL-8 protein release, measured by ELISA, was consistent with expression (LPS AA2 vs AA43 and AA44 p<0.05) (Figure 3B). Similar results were obtained in C38 cells, even though these cells were generally less responsive when compared to IB3-1. LPS from PAO1 was used as positive control and stimulated both IB3-1 and C38 cells (Figure 3A, B and C).

As levels of TNF-α secretion were undetectable in epithelial cell lines, we evaluated LPS stimulation in differentiated macrophagic THP-1 cells (Figure 3D). TNF-α protein release was significantly higher after treatment with LPS from AA2 in comparison to that induced by LPSs from AA43 and AA44 (LPS AA2 vs AA43 and AA44 p<0.05). LPSs from PAO1 and *E. coli* OIII:B4 were used as positive controls and both stimulated THP-1 cells.

### Altered LPS Structure of *P. aeruginosa* Late Strains Induces Low Leukocyte Recruitment and Cytokine Patterns *In Vivo*

**Figure 4:**
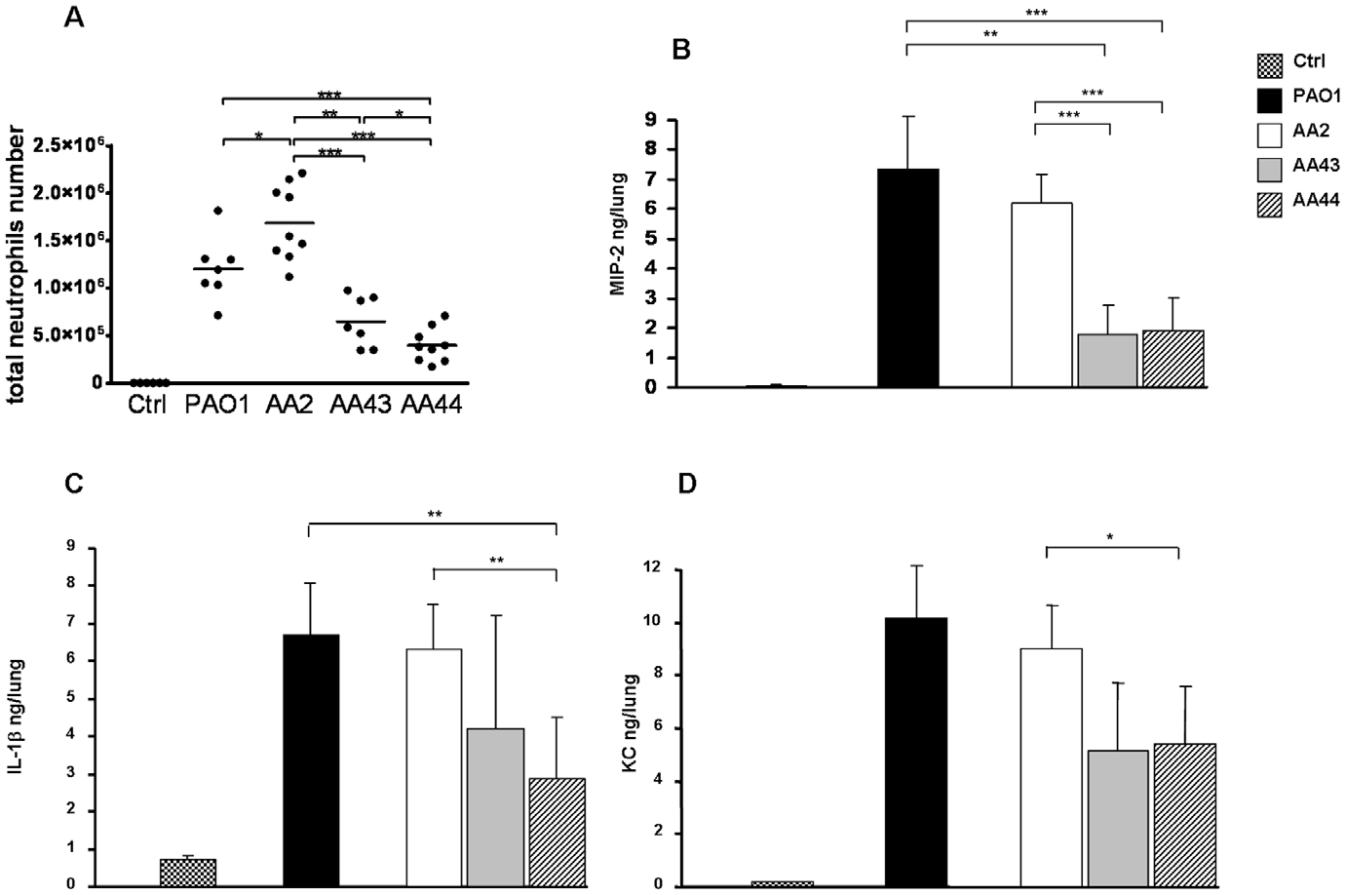
Neutrophils recruitment and cytokines release in murine lungs after 16 h treatment with LPS derived from clinical *P. aeruginosa* strains. C57Bl/6 mouse were exposed for 16 h to 10 mg/mouse of LPS derived from a *P. aeruginosa* reference strain (PAO1) and three clinical isolates (AA2, AA43 and AA44). **A**) Total cells were recovered from the BALF and quantified. **B, C and D**) MIP-2, IL-1β and KC secretions were quantified by ELISA in lung homogenates. *p<0.05, **p<0.01 in the Student's t-test.

To address the question whether the differences in the lipid A structures could affect the inflammatory response *in vivo*, we analyzed leukocytes recruitment in the bronchoalveolar lavage fluid (BALF) of C57Bl/6 mice exposed for 16 h to different LPS structures of strains AA2, AA43 and AA44 by means of nasal instillation. While there was no significant difference in monocytes and lymphocytes number (data not shown), neutrophils profile showed striking differences in total differential cell counts (Figure 4A). Significant higher recruitment of neutrophils was observed in mice exposed to LPS from AA2 strain in comparison to those treated with LPSs from AA43 and AA44 (LPS AA2 vs AA43 and AA44 p<0.001). Cytokines levels were tested in the murine lung homogenates. MIP-2 level in lungs treated with LPS from AA2 strain was significantly higher than those treated with LPSs from AA43 and AA44 strains (LPS AA2 vs AA43 and AA44 p<0.001) (Figure 4B). Similar trends were obtained with IL-1β and KC (Figure 4C and D).

**Figure 5:**
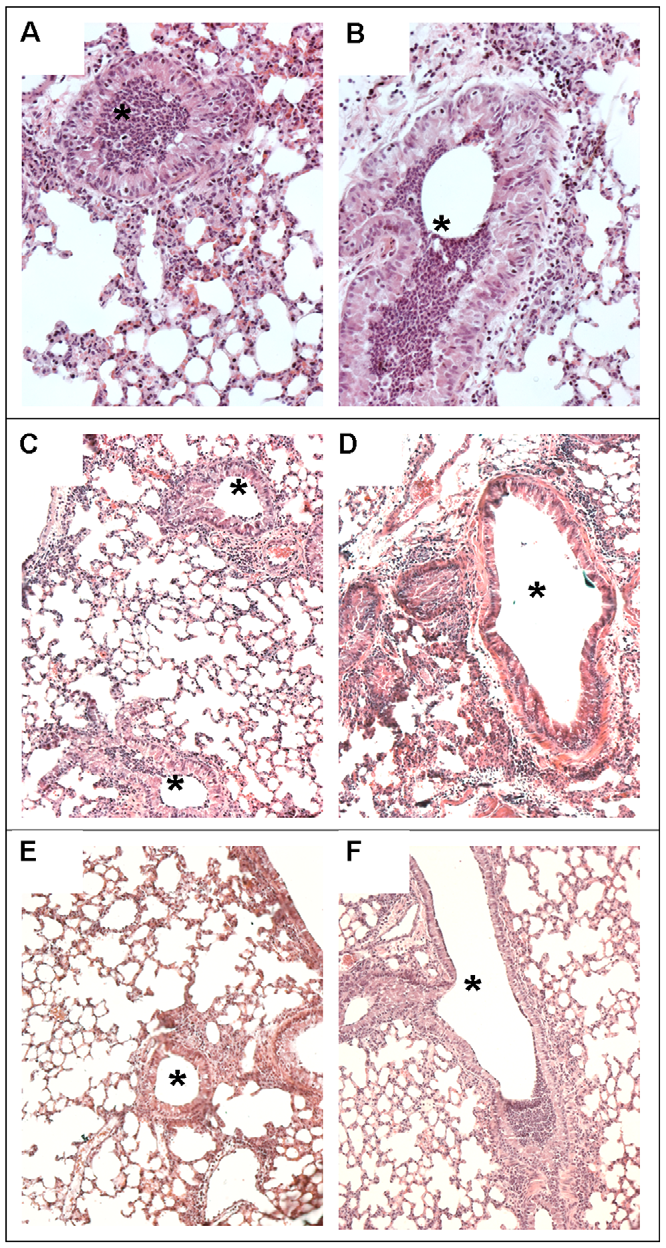
Murine lung histology after treatment with LPS derived from clinical *P. aeruginosa* strains. Mice exposed for 16 h to 10 mg/mouse of LPS derived from three *P. aeruginosa* clinical isolates (AA2, AA43 and AA44). After H&E staining, extensive recruitment of inflammatory cells is visible in the bronchial lumen (asterisk) of animals treated with LPS of AA2 strain (**A and B**), whereas treatment of mice with LPS of AA43 (**C and D**) and AA44 (**E and F**) showed limited accumulation.

Murine lung histopathology showed that the LPS from AA2 strain (Figure 5A and B) caused more severe lesions and leukocytes recruitment in the airways than LPSs from AA43 and AA44 strains did (Figure 5C, D, E and F) indicating a reduced detection for LPS isolated in the late *P. aeruginosa* strains.

### Structural Analysis of *P. aeruginosa* PGN Fragments and Muramyl Peptides in Clonal Strains of Early Colonization and Late Chronic Infection

PGNs from strains AA2, AA43 and AA44 were digested by the muramidase mutanolysin to generate the entire spectrum of muropeptides. The generated PGNs fragments were reduced with sodium borohydride, then identified by RP-HPLC and LC-MS (Figure S3 and S4) [17], [18]. The composition is reported in Table 1 (see Supporting Information File S1 for the whole structure determination).

**Table 1:** Peptidoglycan composition of *P. aeruginosa* strain AA2, AA43 and AA44 after mutanolysin hydrolysis.

AA2	m/z*	% compared to total area of peaks	% compared to more abundant peak
**1-** GlcNAc_1_MurNAc_1_Ala_1_Glu_1_DAP_1_	870	10.4%	40.9%
**2-**GlcNAc_1_MurNAc_1_Ala_1_Glu_1_DAP_1_ **Gly_1_**	927	5.0%	19.7%
**3-** GlcNAc_1_MurNAc_1_Ala_1_Glu_1_DAP_1_ **Lys_1_**	998	7.7%	30.2%
**4-** GlcNAc_1_MurNAc_1_Ala_2_Glu_1_DAP_1_	942	25.6%	100%
**5-** GlcNAc_2_MurNAc_2_Ala_2_Glu_2_DAP_2_	1722	3.7%	14.7%
**6/7-** GlcNAc_2_MurNAc_2_Ala_3_Glu_2_DAP_2_ **Lys_1_/**	1922/	6.9%	26.9%
GlcNAc_2_MurNAc_2_Ala_3_Glu_2_DAP_2_ (eluted in the same peak)	1794		
**7-** GlcNAc_2_MurNAc_2_Ala_3_Glu_2_DAP_2_	1794	4.4%	17.1%
**8-** GlcNAc_2_MurNAc_2_Ala_4_Glu_2_DAP_2_	1865	22.3%	87.1%
**9-**GlcNAc_2_MurNAc(Anh)_1_MurNAc_1_Ala_4_Glu_2_DAP_2_	1845	13.9%	54.3%

AA43	m/z*	% compared to total area of peaks	% compared to more abundant peak
**1-** GlcNAc_1_MurNAc_1_Ala_1_Glu_1_DAP_1_	870	13.3%	42.2%
**2-**GlcNAc_1_MurNAc_1_Ala_1_Glu_1_DAP_1_ **Gly_1_**	927	2.4%	7.5%
**3-** GlcNAc_1_MurNAc_1_Ala_1_Glu_1_DAP_1_ **Lys_1_**	998	7.5%	23.8%
**4-** GlcNAc_1_MurNAc_1_Ala_2_Glu_1_DAP_1_	942	31.5%	100%
**5-** GlcNAc_2_MurNAc_2_Ala_2_Glu_2_DAP_2_	1722	1.2%	3.7%
**6/7-** GlcNAc_2_MurNAc_2_Ala_3_Glu_2_DAP_2_ **Lys_1_/**	1922/	5.7%	18.2%
GlcNAc_2_MurNAc_2_Ala_3_Glu_2_DAP_2_ (eluted in the same peak)	1794		
**7-** GlcNAc_2_MurNAc_2_Ala_3_Glu_2_DAP_2_	1794	2.3%	7.2%
**8-** GlcNAc_2_MurNAc_2_Ala_4_Glu_2_DAP_2_	1865	22.0%	69.5%
**9-**GlcNAc_2_MurNAc(Anh)_1_MurNAc_1_Ala_4_Glu_2_DAP_2_	1845	14.0%	44.4%

AA44	m/z*	% compared to total area of peaks	% compared to more abundant peak
**1-** GlcNAc_1_MurNAc_1_Ala_1_Glu_1_DAP_1_	870	16.0%	65.5%
**2-**GlcNAc_1_MurNAc_1_Ala_1_Glu_1_DAP_1_ **Gly_1_**	927	5.7%	23.5%
**3-** GlcNAc_1_MurNAc_1_Ala_1_Glu_1_DAP_1_ **Lys_1_**	998	7.5%	30.8%
**4-** GlcNAc_1_MurNAc_1_Ala_2_Glu_1_DAP_1_	942	24.4%	100%
**5-** GlcNAc_2_MurNAc_2_Ala_2_Glu_2_DAP_2_	1722	7.0%	28.7%
**6-** GlcNAc_2_MurNAc_2_Ala_3_Glu_2_DAP_2_ **Lys_1_**	1922	1.9%	7.6%
**7-** GlcNAc_2_MurNAc_2_Ala_3_Glu_2_DAP_2_	1794	10.0%	41.1%
**8-** GlcNAc_2_MurNAc_2_Ala_4_Glu_2_DAP_2_	1865	18.2%	74.6%
**9-**GlcNAc_2_MurNAc(Anh)_1_MurNAc_1_Ala_4_Glu_2_DAP_2_	1845	9.1%	37.3%

*mass/charge.

The percentage are based on peak areas derived by HPLC analysis.

Despite the presence of common fragments in the muropeptide blend deriving from the bacterial PGNs, the relative ratio among the peaks sensibly changed among strains AA2, AA43 and AA44 and, furthermore, distinctive chemical features were found in the structure of some minor constituents of the muropeptides mixture (Table 1).

### PGN of *P. aeruginosa* Early Strain Induces Higher Inflammatory Response than Those of Late Strains in Human Cells via Activation of Nod1

**Figure 6:**
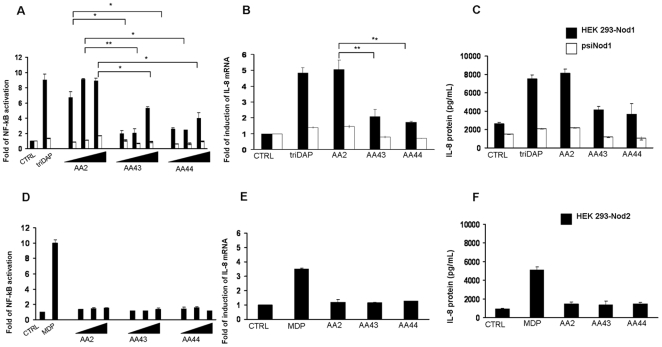
Stimulation of HEK 293-Nod1/psiNod1 and HEK 293-Nod2 with PGN derived from the three clinical isolates of *P. aeruginosa* AA2, AA43 and AA44. **A, D**) Fold of activation of NF-kB after 18 h of stimulation with different concentration of PGN (0,5 µg/mL; 1 µg/mL; 10 µg/mL); triDAP and MDP were used as controls. **B, E**) IL-8 mRNA induction after stimulation with 1 µg/mL of PGN for 18 h. **C, F**) IL-8 secretion after stimulation with 1 µg/mL of PGN for 24 h. *p<0.05; **p<0.01 in the Student's t-test.

HEK293 cells were transfected with hNod1 or hNod2 and stimulated with different concentrations of PGN of AA2, AA43 and AA44 (0.5, 1 and 10 µg/mL) during 18 h. γTriDAP and MDP were used as positive controls. In HEK293hNod1 cells, PGN from AA2 strain induced a major NF-kB activation with respect to PGNs from AA43 and AA44 in a dose dependent manner (PGN AA2 vs AA43 and AA44 p<0.05) (Figure 6A). In contrast with these results no NF-κB activation was found in HEK293 cells expressing hNod2 under the same experimental conditions applied for Nod1 (Figure 6D). IL-8 expression, measured by real time q-PCR in HEK293hNod1 cells treated as above, was significantly higher after stimulation with PGN of AA2 when compared to stimulation with PGNs from AA43 and AA44 (PGN AA2 vs AA43 and AA44 p<0.05) (Figure 6B). IL-8 secretion was measured at 24 h post-transfection. PGN from AA2 strain induced a higher IL-8 production compared to PGNs from AA43 and AA44 strains (PGN AA2 vs AA43 and AA44 p<0.05) (Figure 6C). Transfection of HEK293-hNod1 cell monolayers with a plasmid encoding siRNA for Nod1 prevented the PGNs of the three *P. aeruginosa* strains to activate NF-κB (Figure 6A and B). Likewise, IL-8 expression and production was abolished in the presence of Nod1 siRNA (Figure 6C). None of PGN tested activated IL-8 expression and secretion in HEK-293hNod2 (Figure 6E and F).

**Figure 7:**
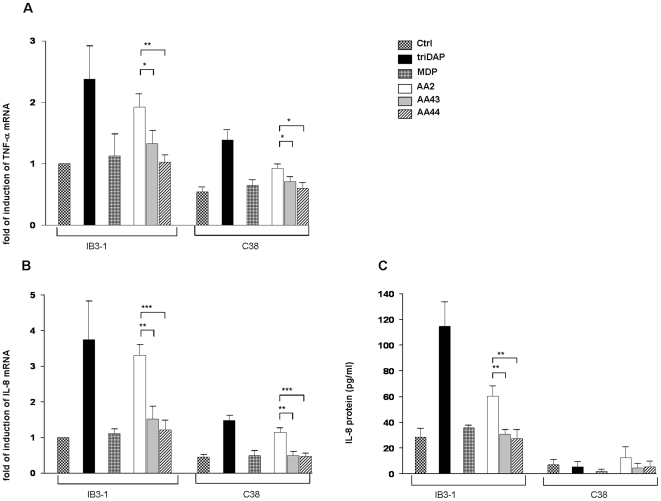
Response of IB3-1 and C38 cells after stimulation with PGN derived from the three clinical isolates of *P. aeruginosa* AA2, AA43 and AA44. **A**) Fold of induction of TNF-α and (**B**) IL-8 mRNA after stimulation with 1 µg/mL of PGN for 4 h. The values represent the expression levels relative to untreated IB3-1 (means±SD). **C**) IL-8 secretion after stimulation with 1 µg/mL of PGN for 24 h. *p<0.05, **p<0.01, ***p<0.001 in the Student's t-test.

Next, Nod1 and Nod2 expression were confirmed in IB3-1 and C38 cells in the absence or presence of stimuli by real time q-PCR (data not shown). TNF-α and IL-8 expression by real time q-PCR was measured at higher levels in IB3-1 cells treated with PGN from AA2 in comparison to PGNs from AA43 and AA44 (PGN AA2 vs AA43 and AA44 p<0.05) (Figure 7A and B). In agreement with these results, levels of IL-8 proteins of cells stimulated with PGN from AA2 were higher than those elicited by PGNs from AA43 and AA44 (PGN AA2 vs AA43 and AA44 PGN p<0.05) (Figure 7C). Similar results were obtained in C38 cells even though these cells were generally less responsive when compared to IB3-1, as showed above for LPS.

## Discussion

In this work, we analysed the PAMPs chemical modification as a strategy of *P. aeruginosa* to hijack genes involved in innate immune responses and to favour survival in patients with CF. A major question, derived from previous reports [7], [8], was whether *P. aeruginosa* could establish life-long chronic infections in CF hosts according with strategies used by a number of other bacterial pathogens. Persistence is normally established after an acute period of infection involving activation of both the innate and acquired immune system. While the acute infection is usually fully resolved by eliminating the invading bacteria, some bacteria including *Salmonella enterica sv typhi*, *Helicobacter pylori*, *Mycobacterium tuberculosis* and others survive and cause persistent life-long infection by evading immune surveillance [19].

Chemical structure of LPS and PGN were determined for three *P. aeruginosa* clones isolated from airways of a CF patient during a period of 7.5 years. Our previous study showed that these *P. aeruginosa* strains, which were isolated at the late stage of CF chronic infection, have better capacity to persist in the murine lung and to cause chronic infection when compared to early strain [8]. Overall, in all three strains LPS lipid A were consistent with the results reported previously though this molecule displayed much more heterogeneous composition than other case-studies [10], [11], [20]. Among the three strains LPS lipid A diversity was observed in the number and location of fatty-acid side chains. Early AA2 and late AA43 mucoid *P. aeruginosa* strains synthesized a LPS blend essentially composed by tetra-, penta- and hexa-acylated species lacking 10∶0 (3-OH) primary fatty acid. In contrast, the late non-mucoid AA44 strain was constituted by homologue lipid A species which further carried a 10∶0 (3-OH) residue, i.e., hexa-acylated and hepta-acylated moieties. These findings are in accordance with previous observations [10], [11], [20] that *P. aeruginosa* synthesizes more highly acylated (hexa- and hepta-acylated) LPS structures during adaptation to the CF airways. Furthermore, our data might be particularly significant as here we compare LPS of a pair of serial *P. aeruginosa* strains, including mucoid and non-mucoid phenotypes. In mucoid AA43 strain the capsule may act as a physical barrier thus partially preventing invasion by host phagocytes and can also mask the LPS molecules to prevent complement deposition, as observed in other microorganisms, such as *E. coli*, *Haemophilus influenzae* and *Klebsiella pneumoniae* [21], [22], [23]. By contrast, for the non-mucoid AA44 the absence of capsule may make this strain more susceptible to phagocytosis. This issue could underline a major selective pressure on AA44 to drastically reduce the immunostimulatory activity of its LPS, promote intracellular survival and resistance to antimicrobial peptides through relevant structural changes in lipid A.

As previously described, *P. aeruginosa* strains with severe CF lung disease lacked deacylated lipid A structures, suggesting that loss of deacylase enzymatic activity (PagL) can occur during long-term adaptation to the CF airway [10], [24]. Retention of the 3-hydroxydecanoic acid at the lipid A 3-O position is presumably due to lack of expression of PagL, a recently identified 3-O position lipid A deacylase [13]. However, genetic data in support of biological activity have not been provided yet. Here, sequence analysis and alignment of the three clonally related *P. aeruginosa* strains demonstrated for the first time pathoadaptive mutation in *pagL* of late AA44 strain. These results indicate that *P. aeruginosa* modified lipid A upon pagL inhibition contributes to reduce TLR4-signalling correlating with data previously obtained for other gram-negative bacteria as *Salmonella typhimurium* [12] and *Bordetella pertussis* [13].

In contrast to AA44, the early AA2 and the late AA43 strains were equipped essentially by penta- and hexa-acylated lipid A but differed in their ratio. The lipid A isoforms have profound implications for human disease, owing to altered recognition by the TLR4 complex [14]. A variety of studies in different bacterial species indicated that the acylation state of lipid A can alter TLR4-mediated responses [25]. In general, production of fully hexa-acylated lipid A is associated with a more strong TLR4 mediated inflammatory response while lipid A with lower levels of acylation triggers reduced cellular responses [25]. This notion has been also demonstrated in *P. aeruginosa* when single species of penta- or hexa-acylated lipid A were tested [11], while in *Salmonella* hepta-acylated isoforms display significantly lower stimulatory activity as compared to the hexa-acylated species [26]. Interestingly, our results suggested that, in addition to the isoforms, also the ratio of penta- and hexa-acylated lipid A had an impact on the biological activity. As shown previously, lipid A under-acylated with respect to hexa-acylated is not only reduced in its ability to stimulate host cellular immune responses, but can also act as antagonist capable of blocking the immunopotential of hexa-acylated *E. coli* LPS lipid A [27], [28], [29], [30], [31]. In particular, penta-acylated lipid A of *P. aeruginosa* could antagonize TLR-4 dependent responses of the human cells to hexa-acylated lipid A from *E. coli* [32].

On the basis of the previous notions it was not surprising that in our study LPS of the late AA43, harbouring a mixture of hexa- and under-acylated lipid A, and AA44, presenting hexa-acylated and hepta-acylated lipid A moieties, displayed a reduced immunomodulatory TLR4-mediated activity compared to LPS of early AA2. In particular, the LPSs of AA43 and AA44 showed weak NF-κB and IL-8 inflammatory response when tested in HEK293 cells expressing TLR-4. Similar results were confirmed in macrophage-like THP-1 cells, in a CF airway epithelial IB3-1cell line and in its isogenic non-CF C38 cells, even though the latter were reported as generally less responsive to stimuli when compared to IB3-1 [33]. In the lung of mice neutrophils recruitment was higher after stimulation with lipid A from early AA2 strain with respect to lipid A from late strains AA43 and AA44. A pool of cytokines including MIP-2, KC and IL-1β were found decreased confirming a reduced inflammation for the LPSs of late strains. Altogether these results emphasize the reduced immunopotential of LPS extracted from late colonizer *P. aeruginosa* strains, in line with other reports that demonstrated the lost of large arsenal of virulence factors during chronic infection [7].

We supported the findings described above on LPS by analysing PGN, as additional structure, in our *P. aeruginosa* strains. The concept that bacteria could modulate PGN composition to escape the immune surveillance has been recently reported for pathogens and commensals but not in *P. aeruginosa* [34], [35], [36], [37], [38]. In accordance with data of lipid A composition, structural analysis of PGN fragments derived by muramidase digestion of PGN from the three clonal strains of *P. aeruginosa* showed a different relative amount of muropeptides.

Despite these differences and in agreement with the biological impact of lipid A of the three clinical isolates, muropeptides of the late AA43 and AA44 strains displayed a dramatic reduced ability to stimulate the Nod1 receptor in HEK293 and CF cells, with respect to PGN of the early AA2 strain. Nod1 and not Nod2 is involved in the NF-κB and IL-8 mediated response to *P. aeruginosa* PGN, as shown in HEK293 cells expressing alternatively these two PRRs or silenced for Nod1. This result is consistent with another previous study [39] that reported the peculiar ability of *P. aeruginosa* to elicit a Nod1-mediated response. At our knowledge, this is a first report that shows an immune evasion strategy involving the *in vivo* selection of pathogenic morphotypes harboring PAMP variants able to modulate recognition by different PRRs.

The minimal structure recognized by Nod1 is the dipeptide D- γ-glutyamyl-meso-diaminolimelic acid (iEDAP) [40] while it is evident that the intact macromolecular PGN does not stimulate Nod1. It was initially described that the Nod1 recognition system requires the exposure of the DAP moiety [40]. Then, other reports, employing synthetic compounds, demonstrated that an exposed DAP moiety is not essential for sensing by Nod1 [41], [42]. However, the identity of major Nod1 stimulatory molecule(s), as well as that of putative antagonistic molecules, remains still unknown.

Therefore, as the molecular details of the PGN-Nod1 relationship are not yet elucidated, we are not able to clarify the mechanisms underlining the reduction of Nod1 activation observed with the PGN of the *P. aeruginosa* strains isolated from the chronic stage of the infection. Difference in muropeptide composition suggests that the enzymatic mechanisms accounting for PGN modifications in AA2, AA43 and AA44 are different or at less differently regulated in these strains. For example, we may observe a reduced amount of GlcNAc_2_MurNAc(Anh)_1_MurNAc_1_Ala_4_Glu_2_DAP_2_ in the PGN of AA44 strain with respect to the other two strains. This might indicate that the activity of the *P. aeruginosa* lytic transglycosylases already identified on the *P. aeruginosa* genome [43] is different in AA44 with respect to the other strains.

However, we might address the question of how the reduced activation of TLR4 and Nod1 could help the persistence of the *P. aeruginosa* strains associated to late stage of CF infection. Actually, the reduced production of inflammatory mediators following activation of TLR4 by LPS of late colonizer strains could account for an impaired ability of the host to mount an adequate reaction aimed at *P. aeruginosa* clearance. Likewise, the role of Nod proteins, and in particular Nod1, has been recently demonstrated in the lung infections sustained by bacterial pathogens, such as *Chlamydophila pneumonia* [44], [45]. In particular, it has been reported that in a murine model of infection the absence of Rip2, which acts as an adaptor molecule in Nod signalling, favors significant delay in *C. pneumonia* clearence from the lungs and promotes more severe and chronic lung inflammation.

Taken together our results, obtained by comparing sequential strains isolated from one CF patient, showed that the synergy of the action of the two modified *P. aeruginosa* PAMPs, PGN and lipid A, could drastically impair the CF host immune detection system thus preventing the eradication of the infection with this pathogen. This failure occurs despite the deleterious consequence of the persistent inflammatory reaction that leads to the decline of lung function and likely to fatal outcome of the disease. These findings emphasize further studies to determine the generality of this mechanism with additional *P. aeruginosa* clonal lineages or to establish whether other bacterial ligands play a critical role in evading immune system thus promoting survival and establishing favourable conditions for chronic persistence in CF patients.

## Materials and Methods

### Ethics Statement

Animal studies were conducted according to protocols approved by the San Raffaele Scientific Institute (Milan, Italy) Institutional Animal Care and Use Committee (IACUC) and adhered strictly to the Italian Ministry of Health guidelines for the use and care of experimental animals.

Research on the bacterial isolates from the individual with CF has been approved by the responsible physician at the CF center at Hannover Medical School, Germany. All patients gave informed consent before the sample collection. Approval for storing of biological materials was obtained by the Hannover Medical School, Germany.

### Bacterial Strains and CF Patient

*P. aeruginosa* clinical isolate AA2 was obtained from sputa or throat swabs from CF patient homozygous for ΔF508 *cftr* mutation attending the Medizinische Hochschule of Hannover, Germany at the onset of chronic colonization. AA43 and AA44 were collected 7.5 years after acquisition and before patient's death. Two intermediate *P. aeruginosa* strains, AA11 and AA12, were also isolated after one year of infection from this patient [8]. Additional clinical data were reported in the online data supplement (Figure S1). Genotypic and phenotypic data of *P. aeruginosa* strains were published previously [8], [46], [47]. In particular, to establish the mucoid phenotype quantitative determination of alginate production by *P. aeruginosa* strains was carried out by carbazole assay as already reported elsewhere [46], [8]. PAO1 was used as reference strain [48]. *P. aeruginosa* was cultured in *Pseudomonas* isolation agar (PIA) or Trypticase Soy Broth (TSB) at 37°C.

### LPS and PGN Extraction and Lipid A and Muropeptide Identification

See Supporting Information File S1.

### Sequence Analysis

One loop of a single *P. aeruginosa* colony, grown on PIA, was processed for DNA extraction using a commercial DNA isolation kit (Qiagen) according to the instructions of the manufacturer. PCR amplifications of the entire *pagL*, *phoP*, *phoQ*, *lpxO1* and *lpoO2* genes were carried out using Taq DNA polymerase (Qiagen). The primers used were detailed in the Online data supplement (Table S1 in Supporting Information File S1). The amplified DNA samples were sequenced by standard automated DNA sequence technology employing the primers described above. The sequence results were compared with the PAO1 sequence (www.pseudomonas.org) by the BLAST programme at the NCBI database (www.ncbi.nlm.nih.gov/blast/) and within the strains of the AA lineage in order to determine the occurrence of sequence variants.

### Cell Cultures and Transfections

The human embryonic Kidney epithelial cell line HEK 293 was grown in D-MEM supplemented with 10% of FBS (both by Cambrex Bio Science). Stably transfected cell line HEK 293-hTLR4/MD2-CD14 (InvivoGen) was cultured in DMEM with 10% FBS and 10 µg/mL Blasticidin-S and 50 µg/mL HygroGold® (both by InvivoGen). HEK293 cells were transiently transfected with PolyFect Transfection Reagent (Qiagen) according to the manufacturer's instructions. For NF-kB studies, the cells were transfected overnight with a reaction mix composed by 1 µL of PolyFect Transfection Reagent (Qiagen), 150 ng of Firefly luciferase reporter constructs, pGL3.ELAM.tk (containing NF-kB promoter sequences), and 15 ng of Renilla luciferase reporter plasmid, pRLTK (as an internal control).

To study Nod_(s)_ activity, HEK293 cells were transfected with 10 ng of hNod1 (pcDNA3-Nod1-FLAG) or 1 ng hNod2 (pUNO-hNOD2a – InvivoGen). Vector plasmids (pcDNA3) were used as controls in all transfection experiments.

### siRNA Assay

siRNA inhibition of Nod1 was carried out as previously described [49] by using psiRNAh-Nod1(InvivoGen). Briefly HEK293 were transfected with 1 ng of plasmid expressing psiRNA or vector control (containing a scramble sequence) together with the plasmid expressing hNod1. Nod1 inhibition was evaluated through western blot analysis with mouse mAbs anti-DYKDDDK (Clone 2EL-1B11, Chemicon International) as previously reported [49].

### Cell Cultures of CF Origin

IB3-1 cells, an adeno-associated virus-transformed human bronchial epithelial cell line derived from a CF patient (ΔF508/W1282X) and C38 cells, the rescued cell line which expresses a plasmid encoding a copy of functional CFTR, have been obtained from LGC Promochem [50], [51]. Cells were grown in LHC-8 media (Biosource) supplemented with 5% foetal bovine serum (FBS) (Cambrex Bio Science). All culture flasks and plates were coated with a solution of LHC-basal medium (Biosource) containing 35 µg/mL bovine collagen (BD Biosciences), 1 µg/mL bovine serum albumin (BSA, Sigma) and 10 µg/mL human fibronectin (BD Bio Science) as described [50].

Cells were seeded at the density of 8×10^4^ cells/cm^2^ for 4 h experiments and 4×10^4^ cells/cm^2^ for the 24 h experiments. The day after cells were treated with 100 ng/mL of LPS derived from the clinical isolates AA2, AA43 and AA44. LPS from *P. aeruginosa* serotype 10^22^ (source strain ATCC 27316) (Sigma) was used as control at the same concentration. In the presence of each LPS for 48 h at 100 ng/mL the cell viability was >95% as determined by Tripan Blue exclusion test. Cells seeded at the density described above were also transfected with 0.5% of PolyFect Transfection Reagent and 1 µg/mL of PGN derived from the clinical isolates AA2, AA43 and AA44. γTriDAP (L-Ala-D-Glu-*meso*-DAP), chemically synthesized by AnaSpec (San Jose, CA), and MDP (MurNAc-L-Ala-D-isoGln) (InvivoGen) were used as positive controls at the concentration of 1 µg/mL. Growth media were collected at the end of incubation, centrifuged and stored at –80°C, while cells were lysed.

### Culture of a Macrophagic Cell Line

The non-adherent human myelomonocytic THP-1 cells, obtained from LGC Promochem, were grown in RPMI-1640 supplemented with 10% FBS. Cells were seeded at the density of 8×10^4^ cells/cm^2^ and allowed to differentiate to macrophage like cells by induction with 60 ng/mL of phorbol myristic acid (PMA) for 36 h, as described previously [52]. The differentiated cells were treated for 6 h with 100 ng/mL of LPS derived from the clinical isolates AA2, AA43 and AA44. LPSs from *P. aeruginosa* serotype 10^22^ and from *E. coli* serotype OIII:B4 (Sigma) were used as controls. Growth media were collected at the end of incubation, centrifuged and stored at –80°C.

### LPS and PGN Stimulations and NF-κB Activity Assessment

For LPS, the day before stimulation HEK 293-hTLR4/MD2-CD14 cells were transiently transfected with the NF-kB reporter constructs as above. Twenty-four hours post-tranfection, cells were left untreated or incubated during 4 (to measure NF-κB and IL-8 mRNA) or 24 (for ELISA) h with different concentration of LPS purified from the clinical isolates AA2, AA43, and AA44. Commercial LPS of *P. aeruginosa* PAO1 (10–50–100 ng/mL) (Sigma) was used as positive controls.

For PGN, HEK 293 cells were simultaneously transfected with (i) hNod1 or hNod2 (ii) NF-kB reporter constructs and (iii) muramidase mutanolysin-digested PGN (Sigma) purified by the three *P. aeruginosa* clinical isolates AA2, AA43, and AA44, at different concentrations (0,5–1–10 µg/mL). γTriDAP and MDP were used as positive controls at the concentration of 1 µg/mL.

NF-kB-dependent luciferase activation and IL-8 mRNA evaluation was measured at 18 h of post-transfection while IL-8 ELISA was performed at 24 h post-transfection.

To measure NF-kB activity, cells stimulated with LPS or PGN, were lysed in 30 µL of passive lysis buffer, and 10 µL of lysate were assayed for Firefly and Renilla activity according to the manufacturer's instructions. The data shown represent the mean values±S.D. of three separate experiments performed in duplicate. Results are reported as fold induction of relative luciferase units (RLU) over unstimulated cells. Relative luciferase activity is calculated as the ratio between the value of the NF-κB inducible Firefly luciferase and that of the constitutive Renilla luciferase reporter.

### RNA Quantification

Total RNA was extracted from lysed cells with the Total RNA Isolation kit (Roche), converted to cDNA with High Capacity cDNA Archive Kit (Applied Biosystems) and random primers and finally amplified using the Platinum^®^ SYBR^®^ Green qPCR SuperMix-UDG (Invitrogen) as described [53]. Primer sets for TNF-α and IL-8 have been reported previously (Sigma-Genosys) [54], [53].

### IL-8 and TNF-α Secretion

Released IL-8 and TNF-α were determined in supernatants collected from the cell cultures using ELISA kits (Biosource Europe and R&D Systems). According to the manufacturer the sensitivities of the assays are less than 0.7 pg/ml for IL-8 and 11 pg/ml for TNF-α. Measurements were performed at least in duplicate. Values were normalized to 10^6^ cells; results were expressed as mean±SD.

### Animals

C57Bl/6 mice (20–22 gr) were purchased by Charles River. Mice were housed in filtered cages under specific-pathogen conditions and permitted unlimited access to food and water. Mice were exposed to intranasal injection of LPS (10 mg/mouse) derived from *P. aeruginosa*. Control animals were exposed to PBS. Sixteen hours after exposure, mice were sacrificed by CO_2_ administration. Bronchoalveolar lavage (BAL) was performed by washing the lungs three times with 1 ml of RPMI (Euroclone) with proteinase inhibitors. Lungs were removed and homogenized in 1 ml of PBS with ions containing proteinase inhibitors. BAL cells were pelletted by centrifugation and counted in a hemacytometer, and differentials were determined by examination of cytocentrifuge slides stained with May Grunwald Giemsa staining (Sigma). The lung homogenates and BALF were centrifuged at 13000 rpm for 30 minutes at 4°C, the supernatants were stored at −20°C for cytokine analysis.

### Measurement of Murine Cytokines

IL-1β, KC and JE concentration in lung homogenates were determined by ELISA (R&D Systems), according to manufacturer instructions using antibody pairs and recombinant standards from R&D System.

### Histology

For histopathology, lungs were removed *en bloc* and fixed in 4% paraformaldehyde/PBS, at 4°C for 24 h, and processed for paraffin embedding. Longitudinal sections of 5 µm taken at regular intervals were obtained using a microtome from the proximal, medial and distal lung regions. Sections were stained with H&E according to standard procedures.

### Statistical Analysis

Statistical calculations and tests were performed using Student's t test considering p≤0.05 as limit of statistical significance. All data were expressed as mean±standard deviation (SD).

## Supporting Information

Figure S1 *P. aeruginosa* sequential isolates from patient AA.
(0.16 MB TIF)

Figure S2 Sequence alignment of *pagL* in sequential *P. aeruginosa* isolates.
(0.93 MB TIF)

Figure S3 RP-HPLC analysis of PGNs fragments.
(0.43 MB TIF)

Figure S4 MS-MS analysis of dimer muropeptide.
(0.16 MB TIF)

Supporting Information File S1 Results concerning full structural assignments of lipid A and muropeptides; experimental procedures; list of primers used for P. aeruginosa gene sequences (Table S1).
(0.08 MB DOC)
