# Supplementary figures and images for: Pseudomonas aeruginosa Exploits Lipid A and Muropeptides Modification as a Strategy to Lower Innate Immunity during Cystic Fibrosis Lung Infection
Source: PLoS One. 2009 Dec 23;4(12):e8439. doi: 10.1371/journal.pone.0008439 (PMC2793027; doi:10.1371/journal.pone.0008439)

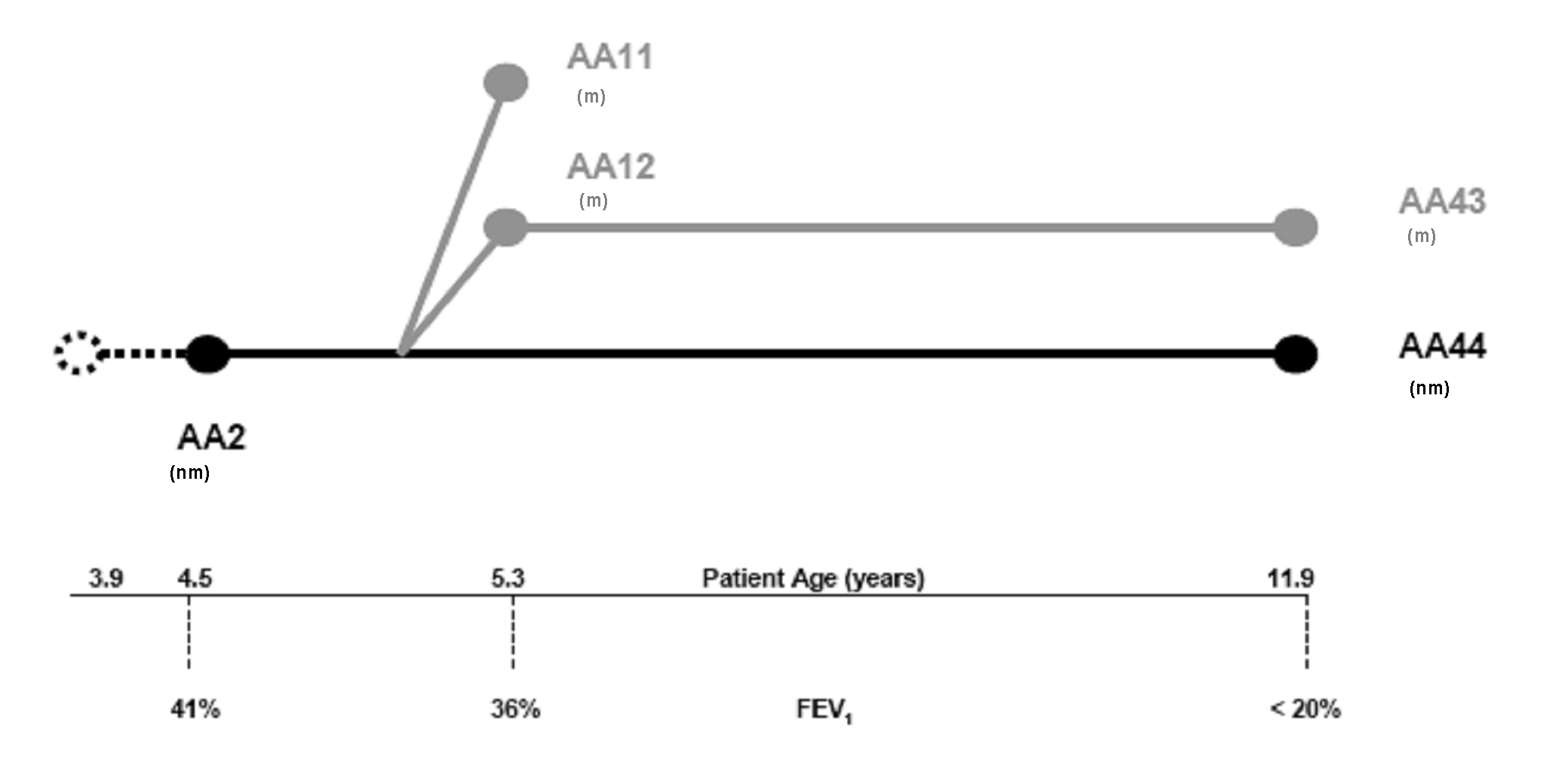

Supplement: Figure S1 — P. aeruginosa sequential isolates from patient AA. (0.16 MB TIF) [file pone.0008439.s001.tif]

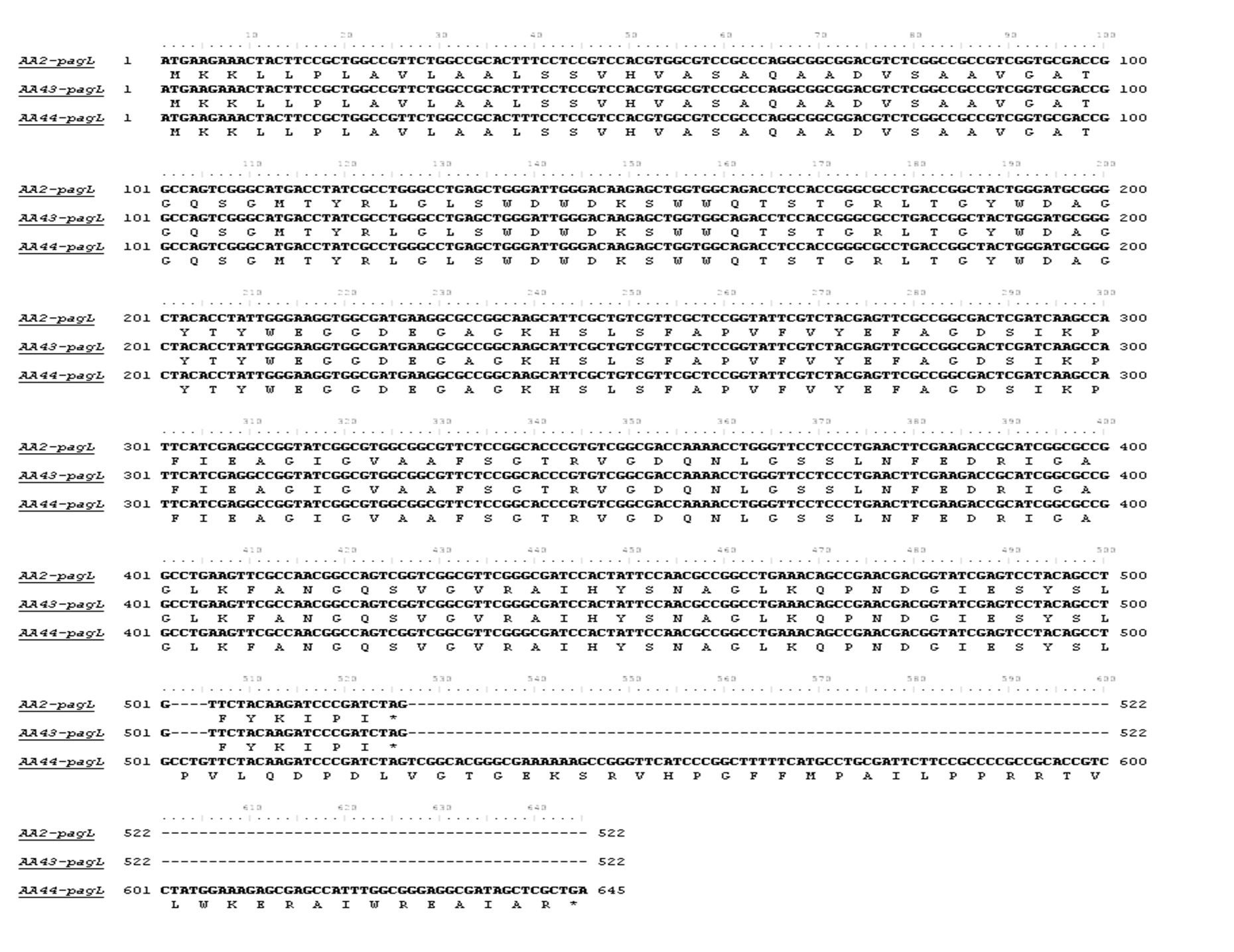

Supplement: Figure S2 — Sequence alignment of pagL in sequential P. aeruginosa isolates. (0.93 MB TIF) [file pone.0008439.s002.tif]

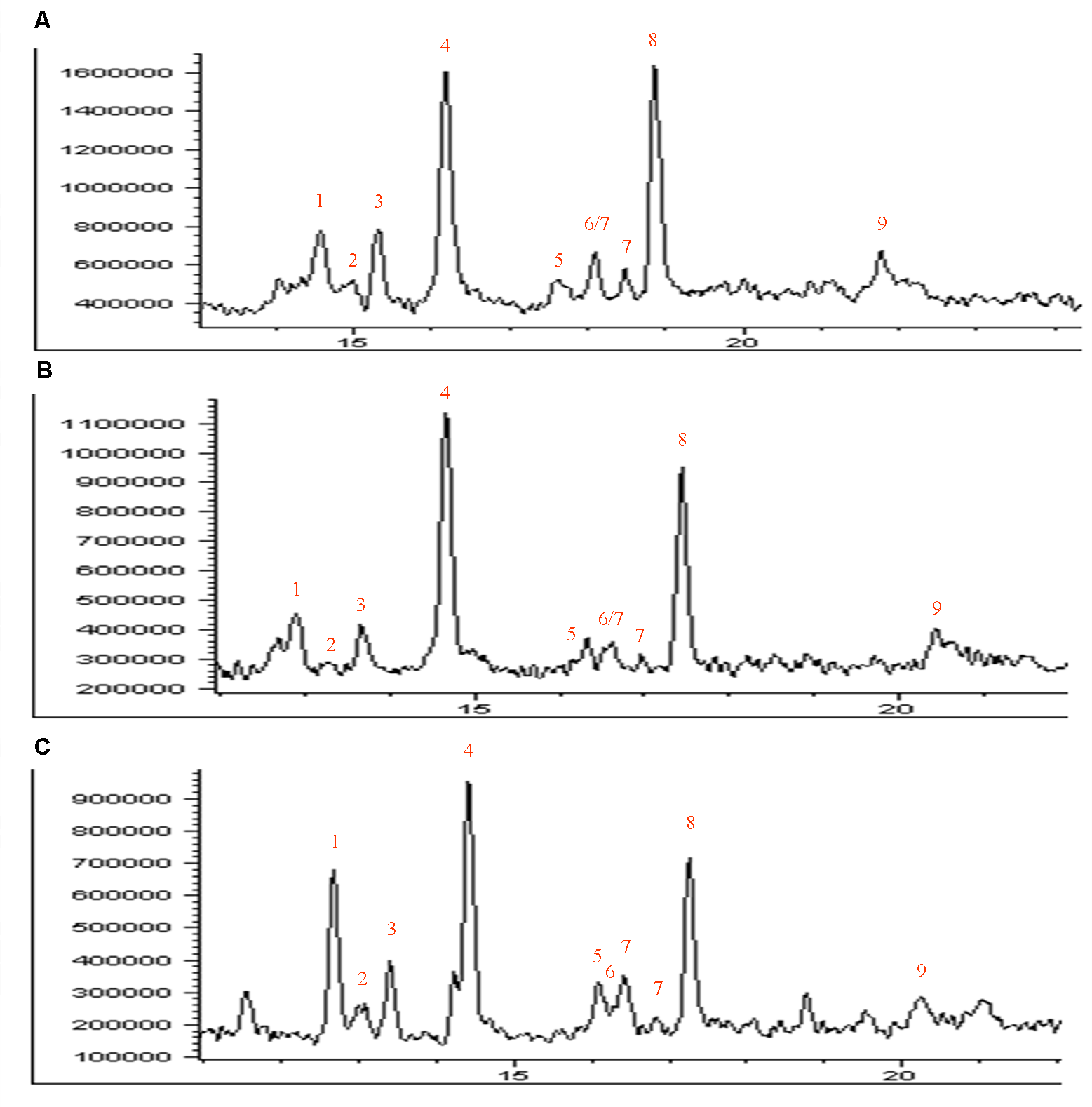

Supplement: Figure S3 — RP-HPLC analysis of PGNs fragments. (0.43 MB TIF) [file pone.0008439.s003.tif]

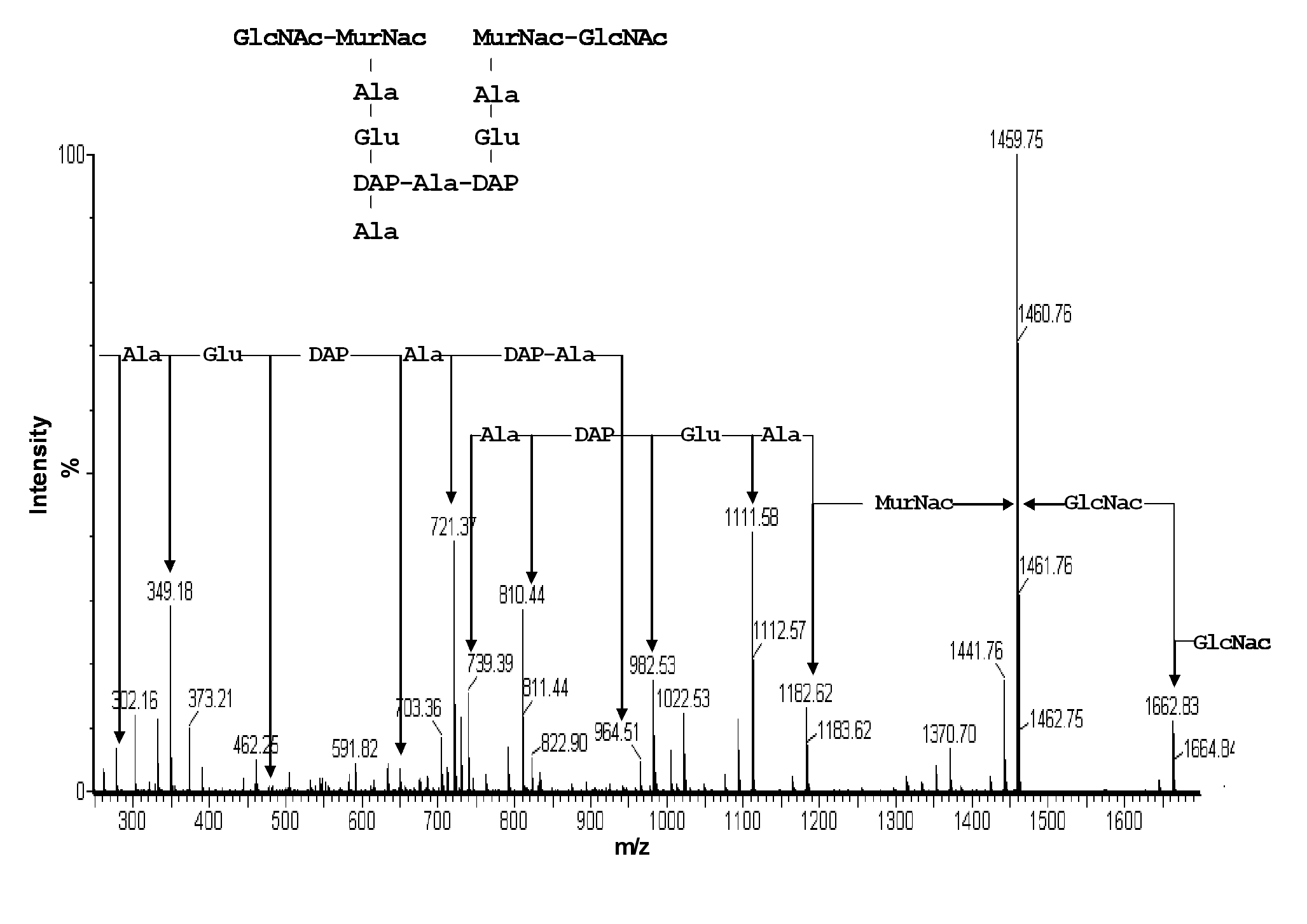

Supplement: Figure S4 — MS-MS analysis of dimer muropeptide. (0.16 MB TIF) [file pone.0008439.s004.tif]
