# Supplementary material for: Pseudomonas aeruginosa Exploits Lipid A and Muropeptides Modification as a Strategy to Lower Innate Immunity during Cystic Fibrosis Lung Infection
Source: PLoS One. 2009 Dec 23;4(12):e8439. doi: 10.1371/journal.pone.0008439 (PMC2793027; doi:10.1371/journal.pone.0008439)
Supplement: Supporting Information File S1 — Results concerning full structural assignments of lipid A and muropeptides; experimental procedures; list of primers used for P. aeruginosa gene sequences (Table S1). (0.08 MB DOC) [file pone.0008439.s005.doc]

***Pseudomonas aeruginosa* exploits lipid A and muropeptides modification as a strategy to lower innate immunity during cystic fibrosis lung infection**

Cristina Cigana, Laura Curcurù, Maria Rosaria Leone, Teresa Ieranò, Nicola Ivan Lorè, Irene Bianconi, Alba Silipo, Flora Cozzolino, Rosa Lanzetta, Antonio Molinaro, Maria Lina Bernardini, Alessandra Bragonzi

Supporting Information

**RESULTS**

**Lipid A structure determination of *P. aeruginosa* clonal strains isolated at the onset of colonization and after years of chronic infection**

Dried cells of the three clinical isolated bacteria were extracted with Westhpal method [51] and a sodium dodecyl sulphate polyacrylamide gel electrophoresis (SDS-PAGE 13.5 %) was performed as described, in order to detect LPS and LOS after staining with silver nitrate. In all three cases, it was exclusively found a LPS fraction in water phases as suggested by the presence of the typical ladder after gel staining. The O-repeating unit resulted identical in all three strains and the structure was already reported [13].

Fatty acids analysis on purified LPS samples revealed the presence of (R)-3-hydroxydodecanoic (C12:0 (3-OH)) in amide linkage and (R)-3-hydroxydecanoic (C10:0 (3-OH)) acid, (R)-2-hydroxydodecanoic (C12:0 (2-OH)), hexadecanoic (C16:0) and dodecanoic acid (C12:0) in ester linkage.

A mild acid hydrolysis was performed in order to isolate lipid A moiety and further investigations, through MALDI MS analysis, were performed on lipid A fractions yielded from this treatment.

MALDI negative ion spectra of the three lipid A examined revealed that LPS extracted from the clone isolated after 6 months of colonization and the one from the mucoid strain isolated after 7.5 years of colonization presented the same lipid A species. In both cases, MALDI mass spectra showed a highest peak that matched with a penta-acylated lipid A. This was constituted by a bis-phosphorylated disaccharide backbone carrying a 10:0 (3-OH) in ester linkage on the β-GlcN and two 12:0 (3-OH) in amide linkage on both GlcN residues. Further, both 12:0 (3-OH) chains were substituted by a secondary fatty acid: on the β-GlcN there was a 12:0 and on the α-GlcN a 12:0 (2-OH). An additional species present was an asymmetric hexa-acylated Lipid A bearing the extra 16:0 that esterifies the 10:0 (3-OH) on the β-GlcN. Species carrying two secondary 12:0 (2-OH) fatty acids and others lacking a phosphate group were also present.

Different species were found for lipid A isolated from the non-mucoid strain sampled after 7.5 years of colonization. The most prevalent species present was a symmetric hexa-acylated lipid A. This was constituted by a bis-phosphorylated disaccharide backbone carrying two 10:0 (3-OH) in ester linkage and two 12:0 (3-OH) in amide linkage. Further, both 12:0 (3-OH) chains were substituted by a secondary fatty acid: on the β-GlcN there was a 12:0 and on the α-GlcN a 12:0 (2-OH). A hepta-acylated lipid A species was also present, bearing the additional 16:0 on the β-GlcN.

**Structure elucidation of PGN fragments, muramyl peptides, released by *P. aeruginosa* clonal strains isolated at the onset of colonization and after years of chronic infection**

PGNs from *P. aeruginosa* strains AA2, AA43 and AA44 were digested by the muramidase mutanolysin to generate the entire spectrum of muropeptides. The generated PGN fragments were reduced with sodium borohydride, then identified by RP-HPLC (Figures 3S and 4S) and LC-MS ([15]). The composition is reported in Table 1 and the primary structure of all PGN fragments was confirmed by ESI-MS-MS experiments.

The spectrum ESI-MS-MS of the more abundant dimeric specie, the muropeptide corresponding to m/z 1865.1 Da, is shown in figure 3S supplemental data.

Following the rapid release of the two terminal GlcNAc residues (ions at m/z 1662.8 and 1459.7), the spectrum is dominated by the y series ions at m/z 1182.6, 1111.6, 982.5, 810.4 and 739.4 corresponding to the loss of MurNAc, Ala, Glu, DAP and Ala respectively. The y series terminated at the m/z 739.4 ion suggesting the occurrence of a branched structure with strong retention of the positive charge that prevented further fragmentation.

Besides the y series, a number of internal fragments could also be detected at m/z 278.1, 349.2, 478.1, 650.3, 721.4 and 964.5 corresponding to the sequence MurNAc-Ala-Glu-DAP-Ala-(DAP-Ala). In particular, the internal ion at 964.5 is diagnostic for the presence of a branched Ala residue attached to the DAP residue..

Despite the presence of common fragments in the muropeptide blend deriving from the bacterial PGNs, unique chemical features were found in the structure of some minor constituents of the muropeptides mixture (Table 1). The compositional analysis of the whole muropeptide fractions showed the occurrence of a glycine (Gly) and lysine (Lys) residues that are unusual in PGN of Gram-negative bacteria.

The structural analysis of the PGN fragments from three clonal strains of *P. aeruginosa* shows not only the presence of common muropeptides generally found in Gram-negative bacteria, but also the unusual fragments with Gly and Lys that are peculiar for the PGN from *P. aeruginosa*. Moreover, the analysis shows also a different relative amount of muropeptides among the three species in accordance with their different biological activity.

**MATERIAL AND METHODS**

**Patients, sample and data collection.** *P. aeruginosa* clones from airways of a CF patient during a period of 7.5 years were selected (Figure S1). This patient was homozygous for the most common CF-causing *CFTR* mutation, ∆F508. Clinical data indicated that he belonged to the subgroup with a severe course of the *P. aeruginosa* airways infection with chronic leukocytosis, elevated serum immunoglobulins and high titers of antipseudomonal IgG in peripheral blood and experienced the most rapid decline of lung function amongst all the more than 600 individuals with CF who had been regularly seen at the CF clinic Hannover since 1980 (Tümmler, personal communication) [8,44].

**LPS extraction**. LPS was extracted by the hot phenol/water procedure as described and revealed by sodium dodecyl sulfate poliacrylamide gel electrophoresis (SDS-PAGE 12%). The gel was stained with silver nitrate for detection of LPSs. In order to get rid of all the cell contaminants, the LPS fraction was further subjected to enzymatic hydrolysis with RNAse, DNAse and proteinase K followed by a size-exclusion chromatography on Sephacryl S-300 in 50 mM NH_4_CO_3_ (yield 170 mg, 1.5% of dried cells).

**Preparation of lipid A.** Free lipid A was obtained by treatment of LPS (10 mg) with 0.1 M sodium acetate buffer (pH 4.4) containing 1% SDS (100°C, 3 h). The solution was then lyophilized, treated with 2 M HCl : EtOH (1:100, v:v) to remove the SDS, evaporated, dissolved in water and ultracentrifuged (100.000 × *g*, 4°C, 90 min). The obtained precipitate (free lipid A) was washed with water (yield: 4.8 mg, 48 % of the LOS).

**Extraction and purification of PGN.** The cell dried are suspended in ice-cold water and added drop by drop to 8% boiling SDS. Samples are boiled for 30 minutes allowing immediate inactivation of autolysins. Polymeric PGN, which is insoluble, is recovered by centrifugation and washed until no SDS could be detected. The complete absence of SDS is tested with Haiashi sage®. SDS treatment removes contaminating proteins, non-covalently bound lipoproteins and LPS. PGNs are further treated with a-amylase to remove any glycogen and with trypsin digestions to remove covalently bound lipoproteins. Samples are further boiled in 1% SDS to inactivate trypsin and washed to remove SDS. Further treatment of PGN includes washes with 8M LiCl, 0.1 M EDTA to remove any polypeptide contaminations and with acetone to remove lipoteichoic acids or any traces of LPS. With this procedure we obtained the PGN with high degree of purity and with a yield of 14,5% for AA2, 3,4% for AA43, 2,3% for AA44.

**Preparation of muramylpeptides and HPLC, LC-MS analysis.** The isolated PGNs were degraded with muramidase mutanolysin from Streptomyces globisporus ATCC21553 (Sigma-Aldrich) at 37°C overnight. The enzyme reaction was stopped by boiling (5 min), and insoluble contaminants were removed by centrifugation. The generated muropeptides were dissolved in 0.5 M sodium borate buffer (pH 9.0), and solid sodium borohydride was added immediately. After incubation for 2 h at room temperature, excess borohydride was destroyed with 2 M HCl. Finally, the samples were adjusted to pH 3–4 with TFA. Reduced muropeptides were fractionated by HPLC by using C-12 reverse-phase column (250×2mm), with 0.05% TFA (solvent A) and 0.05% TFA in 50% acenonitrile (Solvent B). The PGN fragments were eluted with a linear gradient (run time 35 min) from 0% to 60% of eluent B. Detection was performed at 206 nm. ESI-MS spectra were obtained in 2% formic 1:1 acid:methanol (v/v) and 1:1 water:methanol (v/v), respectively (Agilent 1100MSD) (Figures S3 and S4).

**Tandem Mass Spectrometry Analysis.** The PGN fragments were isolated by HPLC on a HP 1100 (Agilent Technologies) by using a Phenomenex narrowbore C18 reverse phase column (250X32 mm) with 0.1% TFA (Solvent A) and 0.07% TFA in 95% acetonitrile (Solvent B) as solvent systems. The sample was eluted by means of a linear gradient from 0% to 20% of Solvent B over the course of 30 min Tandem mass spectral analyses were carried out on a hybrid Q-TOF instrument (Waters) equipped with an electrospray ion source. An aliquot of the HPLC-purified muropeptide was introduced into the mass spectrometer source by direct injection, and a full-scan MS spectrum was acquired. The doubly charged ion corresponding to the muropeptide was isolated into the quadrupole analyzer and fragmented into the collision cell by using argon as the collision gas.

**Table S1 List of primers used for *P. aeruginosa* gene sequences.**

| **Gene** | **PA number** | **Primer** | **Sequence 5’->3’** |
| --- | --- | --- | --- |
| *pagL* | PA4661 | *pagL*-AF | CTTATAATGCGCAGCTTGAATG |
|  |  | *pagL*-AR | CTTCGAAGTTCAGGGAGGAAC |
|  |  | *pagL*-BF | GACAAGAGCTGGTGGCAGAC |
|  |  | *pagL*-BR | AATGGCTCGCTCTTTCCATAG |
| *phoP* | PA1180 | *phoP*-F | CATCTCATCCGGAGGAACC |
|  |  | *phoP*-R | CGCCAGCATGAACAGCAC |
| *phoQ* | PA1179 | *phoQ*-AF | GCCAGGGCTACCTGTTCACC |
|  |  | *phoQ*-AR | CAGCGGCGTCTTCAGACTGT |
|  |  | *phoQ*-BF | GCGAGCAGCTCTACCTGTG |
|  |  | *phoQ*-BR | AAACACCTCAGACGTAGGGAAC |
| *lpxO1* | PA4512 | *lpxO1*-F | GGAGTAAATTCTCCTCTTTTGCAC |
|  |  | *lpxO1*-R | GTGTACTGAACGGATGAATGAAAA |
| *lpxO2* | PA0936 | *lpxO2*-F | CTGATATCGAAGGCATGGAAAG |
|  |  | *lpxO2*-R | CGGGTGTTAATGGGAAAATTAC |
